# Colloid Cyst of 3rd Ventricle
*A case report at a Clinico-Pathological Conference held on 2nd March, 1954, by Prof. T. F. Hewer, in the Department of Pathology, University of Bristol.


**Published:** 1954-10

**Authors:** 


### COLLOID CYST OF 3rd VENTRICLE*

*(A Clinico-Pathological Report)*

*Dr. W. O. Spence:* The patient was a Hungarian and he was a very fit and strong man. He was working as a carpenter although he was actually a horticultural expert. He told me that he had been having headaches for about 6 months before his death in December, 1953, and he had not taken very much notice of them but that they were getting worse. The first intimation I had that anything was wrong was when he came to me complaining of headache and saying that the light bothered him: he was very photophobic. Two days later I was sent for because he had a very severe headache and vomiting. I thought it might be a sub-arachnoid haemorrhage and that it was sufficiently serious to be investigated further, so I sent him to Ham Green Hospital. He was there for two days and was sent home pending further investigations without any definite diagnosis being made but with the very interesting finding of changes in the right fundus: that is, early swelling of the disc. The left fundus was normal. He had recovered completely, resumed his occupation and attended hospital as an out-patient for further investigation. He continued to have headaches and occasional vomiting. I saw him on the 25th November when he had a severe headache and could not stand the light. His headache increased and eventually he was admitted to the Bristol Royal Infirmary. He was quite rational; blood pressure was normal. His disc was still showing optic atrophy and he died after six or eight hours.

*Dr. G. D. W. McKendrick:* I must comment on something I have just learnt from the history. This man denied continuously that he had ever had a headache before the 28th October, and as far as I was concerned his symptoms started four days before he came into Ham Green Hospital.

On admission he had a temperature of 99·8° F. which was the only fever recorded whilst in hospital. There were no abnormal physical signs on examination apart from the right disc which was a little pink with some blurring of the upper and lower margins; the blood pressure was 120/80, the cerebro-spinal fluid normal with 4 cells and 20 mgm. per cent. protein. The pressure was not measured.

The day after admission he was much better and his headache had gone in forty-eight hours. Apart from one small vomit after admission he remained well and did not develop any focal C.N.S. signs. He was very loath to stay in hospital and was sent home with a tentative diagnosis of optic neuritis. He attended Out-patients on the 9th December, when he felt quite well. He still had no focal signs but his right disc was the same. However, he had had one further attack of headache and vomiting which started about a week after he left hospital and lasted ten days. This suggested that his disc changes were due to raised intracranial pressure rather than to optic neuritis, and I referred him to Dr. Campbell.

*Dr. A. M. G. Campbell:* He never got to me. The first time I saw him was on the post-mortem table. Dr. Colson can tell you more than I can.

*Dr. G. Colson:* I can just confirm that he was finally admitted to the B.R.I. on the 13th December, when he was perfectly orientated and stated that he had had these attacks of headache for six weeks. The story here is absolutely as described before. He had had one attack since leaving Ham Green Hospital.

In the ward, Dr. Bain noted that he was a strong man, with marked headache, restlessness and photophobia. There were no abnormal physical signs whatever apart from the right optic fundus; there was no papilloedema, but only some congestion of the vessels. The left fundus was normal. He was thought to have migraine and was

* A case report at a Clinico-Pathological Conference held on 2nd March, 1954, by Prof. T. F. Hewer, in the Department of Pathology, University of Bristol.

given analgesics. A few hours later he complained of headache, vomited three times and suddenly died.

*Professor C. Bruce Perry:* Was his optic atrophy primary or secondary?

*Dr. G. Colson:* Swelling and papilloedema and optic atrophy are mentioned here.

*Professor Perry:* Can anybody tell us what the onset of the headaches was?

*Dr. Colson:* They were gradual.

*Professor Perry:* How long did they take to work up to maximum?

*Dr. Colson:* The headache started on the 28th October and lasted until the 31st October; he vomited at work and went home. I never saw him with severe headache. The position of this headache was frontal, slightly more marked on the right side. He never lost consciousness.

*Professor T. F. Hewer:* Dr. Farrant will now give us the post-mortem findings.

*Dr. P. C. Farrant:* This man was extremely well developed and muscular. I noted that his left pupil was larger than his right. The brain weighed 1,545 grams, which is a bit large; the convolutions were flattened out and, looking at the base of the brain, there was grooving at the bottom of the cerebellum which indicated that some coning had taken place and both tonsils of the cerebellum had been pushed down through the floor of the foramen magnum. There was also slight protrusion of the 3rd ventricle. So here was a rather large and flabby brain. At this point, I asked Dr. Bishton's advice as to whether I should cut it or not. Dr. Bishton took one look at the brain and said that I should make one cut—it might be a 3rd ventricle cyst: it was! There was a colloid cyst of the 3rd ventricle which had been compressing the foramina of Monro laterally causing dilation of both lateral ventricles and hydrocephalus. It arose from a wide but rather tenuous attachment from the roof of the 3rd ventricle: histologically this cyst is filled with colloidal material which before fixation was translucent, and in the colloid material there are scattered groups of inflammatory cells, leucocytes and a few macrophages. The inner lining has a single layer of cuboidal epithelium, in places ciliated. Externally the cyst is lined by rather flattened cells which may be a definite layer or may be due to stretching.

These cysts have been variously described as arising from the choroid plexus, the ependyma or the paraphysis. They only occur in the 3rd ventricle and this fact makes their derivation from choroid plexus and ependyma less likely. On the other hand, choroid plexus during development does grow down from this position to the lateral and the 3rd ventricles. As to the theory that they come from the paraphysis, this is a structure found in certain lower vertebrates—frogs, some fishes and prominently, I believe, in the sturgeon. It is an out-pouching from the front of the 3rd ventricle lined by a derivative of ependyma. In man it is a transient structure which is present only while the embryo is from 19–32 mm. in length. Histology of this particular cyst does not definitely decide the matter but ciliated epithelium is recognizable and the most likely origin is from the paraphysis.

There was another incidental finding: the spleen, thymus and left tonsil were enlarged and I thought it possible that we might be dealing with a case of generalized lymphoid hyperplasia. The lymph nodes, however, were not enlarged. The enlargement of the spleen was due entirely to gross acute congestion. The thymus showed reactive hyperplasia and was, in addition, fatty: it is possible that this could be its normal state in some young healthy adults—this man was 29. The tonsillar enlargement was due to the presence of several hard accretions in its crypts.

So there it is: a case of colloid cyst of the 3rd ventricle with blockage of the foramina of Monro, the development of acute hydrocephalus and death.

*Mr. D. G. Phillips* showed a ventriculogram from another patient which revealed a colloid cyst which was removed successfully and he also showed the cyst itself:

"The cyst is quite simple to remove, approaching it through an opening made in the frontal lobe.

"Alternatively the cyst may be simply coagulated to avoid damaging the large veins in the neighbourhood of the foramina of Monro which might cause severe haemorrhage or infarction.

"While the syndrome of intermittent paroxysmal headache is characteristic, diagnosis on clinical grounds alone is not often made correctly; the ventriculography appearances, however, are pathognomonic."

*Dr. Campbell:* I have little to add except to say that one could not tell on the clinica story this was a 3rd ventricle cyst. There was no temporary weakness of the legs: no focal signs. Why was there a chronic swelling of the disc over this period? There was no high pressure; the disc swelling is confusing.

*Dr. Farrant:* I took the right eye out and on section there was no abnormality in the optic nerve or retina.

